# *Rhodnius prolixus* Viruses Interfere with Proliferation and Metacyclogenesis of the Chagas Disease Agent *Trypanosoma cruzi*

**DOI:** 10.3390/v18030275

**Published:** 2026-02-24

**Authors:** Maira Arruda Cardoso, Carolina Silva Dias Vieira, Isabel Cristina de Faria Moreira, Francis Monique de Souza Saraiva, Ingrid Alexandre de Abreu Brito, Ana Caroline P. Gandara, Rubem F. S. Menna-Barreto, Pedro L. Oliveira, Marcia Cristina Paes, Attilio Pane

**Affiliations:** 1 Instituto de Ciências Biomédicas, Universidade Federal do Rio de Janeiro, Rio de Janeiro 21941-902, Brazil; maira.cardoso@ensino.inca.gov.br (M.A.C.);; 2 Departamento de Bioquímica, Instituto de Biologia (IBRAG), Universidade do Estado do Rio de Janeiro, Rio de Janeiro 20551-030, Brazil; carolina.sd.vieira@gmail.com (C.S.D.V.); isabel.moreira2208@gmail.com (I.C.d.F.M.);; 3 Instituto de Bioquímica Médica, Universidade Federal do Rio de Janeiro, Rio de Janeiro 21941-902, Brazilpedro@bioqmed.ufrj.br (P.L.O.); 4 Laboratório de Biologia Celular, Instituto Oswaldo Cruz, Fiocruz, Rio de Janeiro 21040-900, Brazil; 5 Instituto Nacional de Ciência e Tecnologia em Entomologia Molecular, Rio de Janeiro 21941-902, Brazil

**Keywords:** triatomine, *Rhodnius*, *Trypanosoma cruzi*, virome, RpVs, Chagas disease

## Abstract

The protozoan *Trypanosoma cruzi* is the etiological agent of Chagas disease, a neglected tropical disease that mostly affects the population of Latin American countries, with an estimated 7 million infected people and more than 10,000 deaths per year worldwide. *T. cruzi* is typically transmitted by hematophagous triatomine insects, with *Rhodnius prolixus* being a major insect vector in South America. While the microbiome of triatomine insects has been investigated to a certain extent, the ternary interaction between triatomes insects, *T. cruzi,* and viruses remains virtually unexplored. In this study, we show by transmission electron microscopy and by RT-PCR that *Rhodnius prolixus* viruses (RpVs) can infect the intestine of *R. prolixus*, which places them in close contact with the gut microbiota. These observations suggest that *T. cruzi* can be infected by the insect viruses while transiting through the gut. Here, we show that the RpVs are capable of infecting the epimastigote forms of *T. cruzi* in vitro and maintain the viral load stabilized for 3 to 7 days after infection. We also show that, at least in the case of the iFlavirus RpV1, viral genomes are detectable in the *T. cruzi* cytoplasm. Interestingly, *R. prolixus* ovarian extracts enriched with RpVs decrease epimastigote proliferation and their capacity for differentiation into the ineffective metacyclic trypomastigotes in vitro. Our results start to shed light on the interaction between RpVs and *T. cruzi,* suggesting possible routes of infection and unveiling a role for viral infections in the development of this important pathogen.

## 1. Introduction

Chagas disease is an endemic zoonosis in 21 Latin American countries affecting approximately 6–7 million people and causing about 10,000 deaths per year [1]. The causative agent of this chronic disease is the protozoan *Trypanosoma cruzi*, which is typically transmitted to human hosts by blood-sucking insects of the subfamily Triatominae, chiefly *Rhodnius prolixus* and *Triatoma infestans*, although alternative infection routes have also been reported [2]. The protozoan life cycle begins with the epimastigote form infecting the gut of the insect vector. Epimastigotes differentiate into metacyclic trypomastigote (metacyclogenesis) when they reach the kissing bug hindgut and are then released in the feces. Trypomastigotes can penetrate damaged mammalian skin or mucous membranes, invading the cells, where they transform into amastigote stages with a rounded shape. Amastigotes multiply in mammalian host cells and are released into the bloodstream, where they differentiate into trypomastigotes and can infect the insect during the blood meal [2]. Both trypomastigotes and epimastigotes have an elongated fusiform shape and a flagellum controlling the movement and the attachment to the vertebrate cell surface [3]. While passing through the insect gut, the protozoan comes in close contact with bacteria and viruses composing the insect microbiota [4]. Whether different microorganisms, especially viruses, can impact the development of the protozoan has been poorly investigated. To date, only eight viruses are known to infect triatomine insects. Triatoma virus (TrV) was the first virus identified in Triatomines and shown to belong to the *Triatovirus* genus inside the *Dicistroviridae* family. TrV was shown to cause paralysis of the rear legs and death of *T. infestans* nymphs [5,6]. To our knowledge, the interaction between viruses and *T. cruzi* is still in its infancy. Virus-like particles (VLPs) have been found by transmission electron microscopy in clusters close to the Golgi apparatus from *T. cruzi* epimastigotes but not in metacyclic trypomastigote and amastigote forms [7]. VLPs found in some *T. cruzi* epimastigotes have not been associated with specific viral species, although their non-enveloped capsid and features suggested they might belong to the Iflaviridae family. Recently, our research group has identified and characterized seven new viruses in *Rhodnius prolixus* (RpV1–7) through meta-transcriptomic approaches [8,9]. The viruses belong to three families: Iflaviridae (RpV1 and RpV2), Permutotetraviridae (RpV3, RpV4, and RpV7), and Solemoviridae (RpV5 and RpV6) [8,9], and all of them have (+) ssRNA genomes. We showed that the RpVs can establish persistent infections in insect colonies, which are sustained by vertical transmission via a transovarian mechanism. An RNAi-based antiviral system is active in the insect and likely contributes to maintaining the viral load below lethal levels [9]. These observations strongly suggest that the protozoan *T. cruzi* may come into contact with the insect virome [10] as it transits the insect gut and is ultimately released in the feces. In this study, therefore, we aimed at characterizing the interaction between the RpVs and *T. cruzi* in culture and assessing their impact on the life cycle of the parasite.

## 2. Methods

### 2.1. Sources and Rearing Methods of R. prolixus and T. cruzi

Insects were reared at 28 °C and 70–80% relative humidity and fed with rabbit blood at regular intervals to promote oogenesis [11]. Ovaries were dissected from adult females 7 days after the first or second blood meal. All animal care and experimental protocols were performed according to the ethics guidelines. Husbandry of the rabbits was performed by trained technicians at the animal facility of the Instituto de Bioquímica Médica Leopoldo de Meis (UFRJ), following stringent guidelines and under the supervision of licensed veterinarians. Experimental protocols involving insects were performed in agreement with the guidelines of the Committee for Evaluation of Animal Use for Research (Universidade Federal do Rio de Janeiro, CAUAP-UFRJ) and the NIH Guide for the Care and Use of Laboratory Animals (ISBN 0–309–05377–3). Protocols were approved by CAUAP-UFRJ under registry #IBQM155/13.

### 2.2. Ultrastructural Analysis

*R. prolixus* anterior midguts were dissected and fixed with 2.5% glutaraldehyde in 0.1 M sodium cacodylate buffer (pH 7.2) at room temperature (~25 °C) for 1 h and then post-fixed in 1% OsO_4_ in the same buffer containing 0.8% potassium ferricyanide and 2.5 mM CaCl_2_ for 1 h at 25 °C. Samples were dehydrated in an ascending acetone series and slowly embedded in PolyBed 812 resin (Polysciences lnc., Warrington, FL, USA, five steps acetone/resin: 1:3, 1:1, 1:2, 2:3, and pure resin, each step for 24 h). Ultrathin sections were stained with uranyl acetate and lead citrate and examined in a Jeol JEM1011 transmission electron microscope (Tokyo, Japan) at Plataforma de Microscopia Eletrônica in Fundação Oswaldo Cruz.

### 2.3. In Vitro Proliferation and Metacyclogenesis Assays

*T. cruzi* epimastigote (Dm28c) were infected for 2 h with a bacteria-free supernatant from ovaries of *R. prolixus* macerated and naturally enriched with RpVs. The extract is centrifuged and filtered through a 0.22 µm filter to remove ovarian debris and leave a clearer solution. The viral titers are determined by absolute quantification by RT-qPCR, as we have previously reported [9]. In order to avoid batch effects, we pooled the extracts obtained from 30 adult females dissected 7 days after blood-feeding. The samples were aliquoted in 200 µL batches that were kept at −20 °C. The viral titer ranges from 10^6^ for RpV5 and RpV6 to 10^9^ genome copies per microgram of total RNA for RpV1 and RpV4. RpV2 is not detected in the samples as we previously reported [9]. The experimental protocols and kissing bug handling followed the guidelines of the UFRJ Animal Use Committee (CAUAP-UFRJ, registration number: #IBQM 155/13) and the NIH Guide for the Care and Use of Laboratory Animals. All the infection experiments using RpV-enriched supernatant were conducted in vitro. Epimastigotes (2.5 × 10^6^ parasites/mL) were cultured at 28 °C for 7 days in Brain Heart Infusion (BHI) medium supplemented with 10% FBS and antibiotics (penicillin and streptomycin) after a 2 h pre-incubation in the presence or absence of RpV-enriched extract. Parasite growth was monitored by cell counting using a Neubauer chamber. The proliferation curve was plotted using the GraphPad Prism 8 software. Metacyclogenesis was induced following the protocol described by Contreras and coworkers [12]. Epimastigote forms (5 × 10^8^ cells/mL) were incubated for 2 h in TAU medium (containing 190 mM NaCl, 17 mM KCl, 2 mM MgCl_2_, 2 mM CaCl_2_, 8 mM phosphate buffer, pH 6.0) in presence or absence of the RpVs-enriched solution at 28 °C. Samples were then diluted 100 times in TAU 3AAG (TAU supplemented with 10 mM L-proline, 50 mM sodium glutamate, 2 mM sodium aspartate, and 10 mM D-glucose). Parasites were maintained under these conditions at 28 °C for 96 h. The developmental stages were determined based on their morphology and kinetoplast position after staining with Panotic (Laborclin Ltda, Pinhais, Paraná, Brazil). Images were acquired using an Olympus BX51 microscope (Tokyo, Japan).

### 2.4. RT-PCR and RT-qPCR Assays

Total RNA from the entire gut of *R. prolixus* (including crop, midgut, and hindgut) was extracted using the TRIzol™ protocol (Thermo Fischer, São Paulo, Brazil). cDNA was synthesized using MultiScribe™ Reverse Transcriptase, and the cDNA was loaded in 2% agarose gel. *T. cruzi* epimastigotes (1 × 10^7^ cells/mL) were incubated with RpV-enriched solution for 2 h. After incubation, the parasites were centrifuged and maintained in a renewed BHI medium supplemented with 10% FBS and heme (30 μM) at 28 °C for 2 h, 3 days, and 7 days. After each time point, parasites were washed with PBS, and total RNA was extracted using the TRIzol™ protocol. RT-qPCR conditions and specific oligonucleotides have been previously described [9]. RT-qPCR was performed on the QuantStudio 7 Flex (Thermo Fisher, São Paulo, Brazil), and the data were analyzed by absolute quantification through a standard curve. The standard curve was generated using a 747-nucleotide-long cDNA fragment corresponding to a portion of RpV1 previously transcribed in vitro. RpV1 cDNA (50 ng) was serially diluted from 1:10 to 1:10^8^ to generate the standard curve represented by the linear regression of the Cycle Threshold (CT) values for the RpV1 fragment versus the logarithm of viral RNA molecules (RpVs 1, 4, 5, 6, or 7) per μg of total RNA. The number of RNA molecules of the RpV1 fragment was calculated using the formula $G=\frac{m\times A}{L\times 10^{9}\times Da}$, where *G* is the number of RNA molecules, *m* is the amount of single-stranded RNA (ssRNA) in nanograms, *A* is Avogadro’s constant (6.022 × 10^23^), *L* is the length of the ssRNA in nucleotides (747), and *Da* is the average weight of a single-stranded DNA molecule in Daltons (330) [13,14]. The resulting values were used to calculate the logarithms of the number of RNA molecules per microgram. Based on this standard curve, the number (*N*) of viral genome copies per microgram of total RNA for each virus (RpVs) was calculated as *N* = 10$\frac{CT-b}{m}$, where *CT* is the cycle threshold for each RpV, *b* is the y-intercept of the standard curve, and *m* is the slope of the standard curve. Finally, the number of copies was normalized by the viral genome size [9].

### 2.5. Fluorescent In Situ Hybridization (FISH)

For the synthesis of digoxigenin (DIG)-labeled probes used in FISH, the PCR product amplified using specific primers for RpV1 was purified using the Monarch^®^ PCR & DNA Cleanup Kit (New England Biolabs, NEB, Ipswich, MA, USA). The purified product was used as a template for a second PCR reaction in which a forward primer specific for RpV1 and a reverse primer specific for RpV1 containing the T7 RNA polymerase promoter sequence at its 5′ end were added. The amplicon from the second PCR was used as a template for in vitro transcription of the RpV1 antisense RNA probe. The products of the second PCR were purified with the Monarch^®^ PCR & DNA Cleanup Kit and used for in vitro transcription reactions using T7 RNA polymerase from NEB. The probes were precipitated by lithium chloride (LiCl) and absolute ethanol and resuspended in 50 μL of hybridization solution (50% formamide, 0.1% Tween 20, 1 mg/mL salmon sperm DNA, 5X SSC [3.0 M NaCl and 0.3 M sodium citrate, pH 7.0], 50 μg/mL heparin, and RNase-free H_2_O). Epimastigote forms of *T. cruzi* were infected as previously described and centrifuged at 3500 rpm for 5 min. The epimastigote pellet was carefully washed three times with 1X PBS and resuspended in 400 μL of 1X PBS. Parasite cells (2.5 × 10^6^ cells) were applied to coverslips previously treated with 0.1% Poly-L-lysine at the bottom of a 24-well plate and fixed with 4% formaldehyde (Merck, São Paulo, Brazil) for 20 min and then washed with sterile 1X PBS. To permeabilize the parasites, they were incubated in 70% ethanol at 4 °C overnight and washed with 1X PBS. Pre-hybridization was performed at 55 °C for 1 h with the hybridization solution (50% formamide, 0.1% Tween 20, 1 mg/mL salmon sperm DNA, 5X SSC, 50 μg/mL heparin, RNase-free H_2_O). Then, the coverslips were incubated in the hybridization solution containing the RpV1-antisense probe in a humidity chamber at 55 °C overnight. The probe solution was discarded, and the post-hybridization step was performed at 55 °C for 30 min. The coverslips were washed three times with 0.1% Triton X-100 in 1X PBS for 10 min at room temperature. The coverslips were incubated in a 20% Western Blocking Reaction (Roche, São Paulo, Brazil) for 30 min at room temperature. FISH assays were performed as previously described [15,16]. The antibodies used were sheep anti-DIG 1:1000 (Roche, cat. Number: 11333089001) and Alexa Fluor 594 donkey anti-sheep 1:1000 (Invitrogen, cat. Number: A-11016, São Paulo, Brazil). DAPI 1:1000 (Invitrogen, cat. Number: D1306) was used for DNA staining. Mock parasites were not exposed to the ovarian extracts. They were incubated only with BHI medium. Images were captured using a confocal microscope (SPE, Leica, São Paulo, Brazil).

## 3. Results

### 3.1. Transmission Electron Microscopy Analysis Reveals Viral Particles in R. prolixus Anterior Midgut

**Figure 1:**
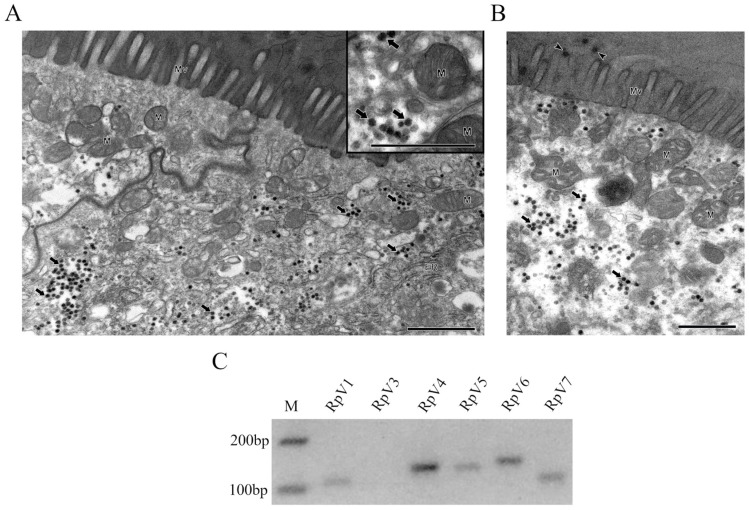
Distribution of the RpVs in *Rhodnius* gut. (**A**,**B**) Ultrastructural analysis of *R. prolixus* anterior midgut revealed the intense virus replication in this tissue (**A**) and viral-like particles in the lumen (**B**). Solid arrows: cytoplasmic viral particles; arrowheads: viral-like particles in the gut lumen; ER: endoplasmic reticulum; M: mitochondria; Mv: microvillus. Bars = 1 µm. (**C**) RT-PCR assays in *R. prolixus* whole gut tissue with oligonucleotides specific to the RpVs. The size of the amplicons is consistent with the expected region of the viral genomes. M: DNA molecular ladder.

Our group initially identified the RpVs in *R. prolixus* ovaries using metatranscriptomic approaches [9]. Thus, we first aimed at investigating whether these viruses can also infect the gut of the insect. An ultrastructural approach was performed to answer this question. Interestingly, VLPs appear to be abundant in the insect gut (Figure 1A). Interestingly, viral-like particles are also detected in proximity to the gut villi and within the intestinal lumen (Figure 1B). In order to confirm their identity, we then performed an RT-PCR assay from *R. prolixus* gut samples, which included the foregut, midgut, and hindgut regions (Figure 1C). The primer pairs used in this assay have been previously described [9]. This approach confirmed that the viruses detected by transmission electron microscopy were the Iflavirus RpV1, the Permutoteraviruses RpV4 and Rpv7 and the Solemoviruses RpV5 and RpV6 [7]. We did not detect the RpV3 virus and, as expected, RpV2, which has been lost from the colony sometime in the past ten years. Taken together, these observations strongly indicate that *T. cruzi* might encounter the RpVs while transiting through the gut lumen of *Rhodnius*.

### 3.2. RpVs Are Capable of Infecting Epimastigote Forms of T. cruzi

**Figure 2:**
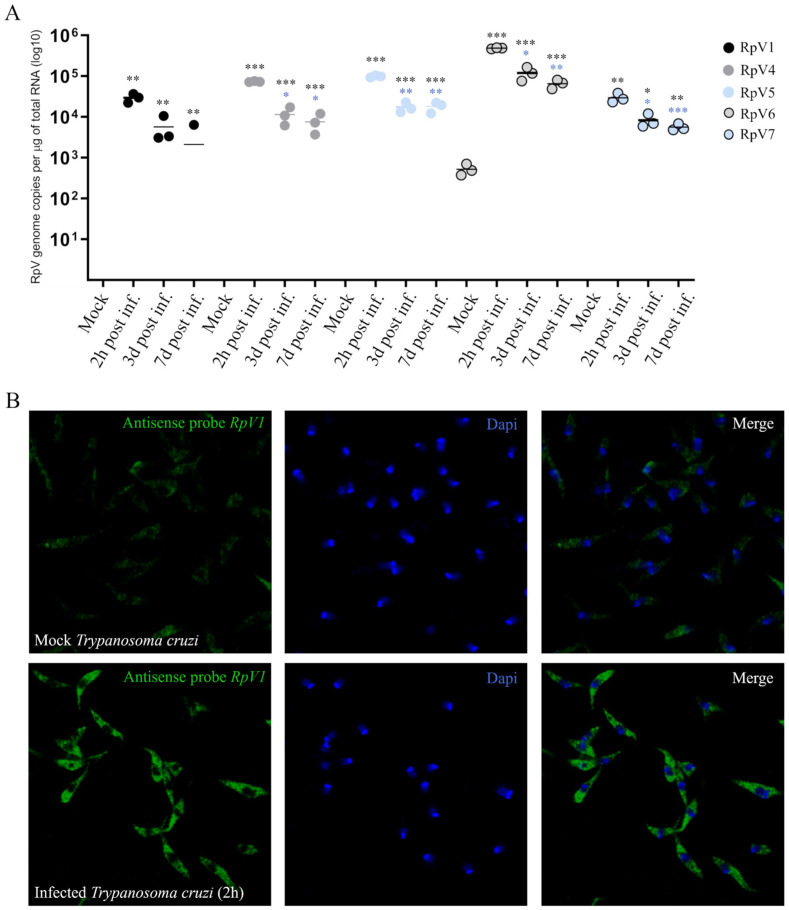
Analysis of the viral genomes in *T. cruzi*. (**A**) Viral load of RpVs in epimastigote forms of *T. cruzi* pre-incubated for 2 h with *R. prolixus* ovary extract and evaluated at 2 h, 3 days, and 7 days post-infection. Statistical analysis of the viral load of RpVs was performed using Student *t* test. * *p* < 0.05, ** *p* < 0.01, and *** *p* < 0.001 compared to the control. Colored asterisks indicate *p*-values corresponding to the colors of each RpV relative to the experimental condition of 2 h incubation with the extract. Data were normalized in log10. (**B**) FISH images of epimastigote forms of *T. cruzi* using a RpV1 antisense probe. Top panels display the control (Mock), while the results of the assay in infected cells are shown in the lower panels. Blue: DNA; Green: RpV1 probe.

We then asked whether the RpVs can infect epimastigote forms of *T. cruzi* in vitro. To this aim, we monitored the viral genome copy number in the epimastigotes exposed for 2 h to the RpV-enriched ovarian extracts. We performed RT-qPCR assays at 2 h, 3 days, and 7 days post-infection. We obtained viral genome copy numbers ranging from ~10^4^ for RpV7 to ~10^6^ for RpV6. The viral load drops by 2 orders of magnitude at 3 days post-infection, suggesting that the virus does not efficiently replicate in the protozoan cytoplasm or that an antiviral system might be counteracting the infection. However, the viral load does not seem to change dramatically over the next four days (Figure 2A). Although we did not investigate the production of viral progeny, these observations suggest that the epimastigote forms of *T. cruzi* may be permissive to RpVs, which can persist in the parasite for several days. This time window might be sufficient for the viruses to modulate the life cycle of the parasite in vivo.

We then asked whether viral genomes can be observed in the cytoplasm of the parasite. To answer this question, we monitored the subcellular distribution of the RpV1 virus, one of the most abundant RpVs in the *R. prolixus* ovary [9], in epimastigote forms of *T. cruzi* (Dm28c) incubated with RpV-enriched ovarian extracts. In the FISH assays using antisense RNA probes for RpV1, we found that 2 h after incubation with the ovarian extract, RpV1 was spread throughout the parasite’s cytoplasm. Differently, a much weaker signal, likely corresponding to background fluorescence, is observed in control cells (Figure 2B).

### 3.3. RpVs Affect Epimastigote T. cruzi Proliferation and Metacyclogenesis

**Figure 3:**
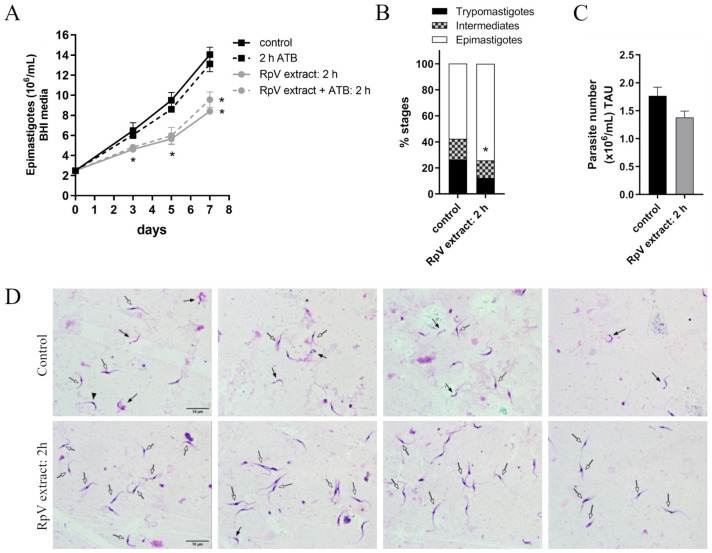
Epimastigote phenotypes induced by incubation of *T. cruzi* with RpV-enriched extract. (**A**) Proliferation of epimastigote forms of *T. cruzi* (Dm28c) infected with RpVs for 2 h in the presence or absence of antibiotics. (**B**) Percentage of metacyclic trypomastigote, intermediates, and epimastigote forms and (**C**) total parasite number in metacyclogenesis following pre-incubation with RpV-enriched extract for 2 h. (**D**) Representative images of *T. cruzi* metacyclogenesis showing four random fields of control parasites or parasites pre-treated with RpV-enriched extract for 2 h. The parasite stages were identified based on morphology and kinetoplast position: metacyclic trypomastigotes (solid arrows), intermediate forms (arrowhead), epimastigotes (open arrows). Graphs show mean ± standard error from at least three independent experiments. Statistical analysis of the percentage of metacyclic trypomastigotes was performed using Student’s *t*-test. For proliferation data, statistical analysis was performed using One-Way ANOVA and Tukey’s post-test. * *p*-value < 0.05 compared to the control.

Finally, we investigated the impact of the RpVs infection on *T. cruzi* development by performing a proliferation assay on epimastigote forms (Dm28c) exposed to RpV-enriched ovarian extract (Figure 3A). We observed a significant decrease in the proliferation rate at 5- and 7-days post-infection compared to the control (Mock), suggesting that RpVs interfere with the parasite’s proliferation rate (Figure 3A). Treatment with antibiotics results in a similar decrease, thus demonstrating that the observed phenotype is not caused by bacteria.

Upon entry into the insect gut, the protozoan undergoes a developmental differentiation from the proliferative epimastigote to the metacyclic trypomastigote form. We observed that pre-incubation for 2 h with RpV-enriched extract significantly decreased (~53%) the differentiation of *T. cruzi* into metacyclic trypomastigotes compared to the control (Figure 3B). Finally, when we counted the number of parasites in treated versus control conditions, we did not observe a significant difference between the *T. cruzi* forms (Figure 3C). Representative images of *T. cruzi* metacyclogenesis showed that control parasites presented a higher number of metacyclic trypomastigote forms, whereas pre-incubation with RpV-enriched extract resulted in most parasites remaining in the epimastigote form after 96 h of differentiation (Figure 3D). These observations indicate that infection with the RpVs in a TAU medium specifically impairs *T. cruzi* differentiation without affecting total parasite number.

## 4. Discussion

In this study, we investigated the interaction between the protozoan *T. cruzi* and insect-specific viruses that we recently identified in *R. prolixus*. Previously, it was shown that TrV, a dicistrovirus isolated from *T. infestans*, appears to favor the infection of *T. cruzi,* probably by increasing the ability of the virus to adhere to the insect gut [17]. However, aside from this study, the interaction of insect viruses with *T. cruzi* has not been explored yet. Here, we show that the RpVs are able to infect the insect anterior midgut and accumulate in the cytoplasm of the cells. Interestingly, we also observed viral-like particles in the gut lumen. This prompted us to investigate whether the RpVs can infect *T. cruzi*, since the epimastigote form is likely exposed to viral infections while transiting through the insect gut. We show that the genome of RpV1, belonging to the iFlavirus family, can be detected in the cytoplasm of epimastigotes and that all the RpVs can persist in the protozoan cells. Sustained high viral copy numbers are, in fact, observed up to seven days after the incubation with RpV-enriched ovarian extracts. Our observations are in agreement with the results by Fernández-Presas and coworkers, who showed by ultrastructural studies that enveloped and non-enveloped viral particles can be observed in the cytoplasm of *T. cruzi* [7]. Notably, the authors concluded that the characteristics of some viral particles were compatible with the structure of IFlaviruses, a family of viruses that includes RpV1 [9]. Following exposure to the virus-enriched extract, we observed a substantial reduction in epimastigote proliferation at 5- and 7-days post-infection, suggesting that one or more RpVs might interfere with the proliferation of the parasites. It has already been shown that *T. cruzi* epimastigotes can be infected by the Triatoma virus (TrV), while this virus was not observed in trypomastigotes or amastigotes [7]. Our results seem to lend support to the conclusion that viral infections, including those involving TrV and RpVs, occur when *T. cruzi* is in the replicative epimastigote form. Finally, our study reveals that the RpVs might also be able to affect the differentiation of the parasite by inhibiting the progression to metacyclogenesis, thus reducing the number of infective trypomastigote forms. It is tempting to speculate that the persistence of viral infections might induce a chronic stress state that interferes with the signaling pathways and gene regulatory mechanisms required for metacyclogenesis. For instance, viral genomes might compete with endogenous transcripts for RNA-binding proteins, translation factors, or organelles, like stress granules. Because the regulation of gene expression in *T. cruzi* is largely post-transcriptional, the persistence of an RNA viral genome within the cytoplasm of the parasite might interfere with the expression or activity of proteins required for the differentiation program. This area of research is still in its infancy, and the present study represents a first step toward a better understanding of the ternary interactions among triatomine insects, viruses, and the Chagas disease agent *T. cruzi*. These findings may pave the way for the use of RpVs or other viruses as biological tools to reduce *T. cruzi* infections in natural populations of triatomine vectors and, consequently, limit the spread of Chagas disease.

## Data Availability

The original contributions presented in this study are included in the article. Further inquiries can be directed to the corresponding authors.
